# The powerful "lens" of magnetic resonance imaging in the diagnosis of hepatic nodules in patients with cirrhosis: diagnosis of hepatocellular carcinoma without the need of histopathological confirmation - fact!

**DOI:** 10.1590/0100-3984.2017.50.1e2

**Published:** 2017

**Authors:** Andrea Farias de Melo-Leite

**Affiliations:** 1 PhD, MD, Residency Preceptor at the Instituto de Medicina Integral Professor Fernando Figueira (IMIP), Radiologist at the Centro Diagnóstico Lucilo Ávila Júnior (CLA), Maximagem, and Safelaudos Diagnósticos, Recife, PE, Brazil. E-mail: andreafariasm@gmail.com.

Chronic liver inflammation, which is responsible for various stages of parenchymal fibrosis and, especially end-stage liver disease (cirrhosis), is the main cause of hepatocarcinogenesis and therefore of hepatocellular carcinoma (HCC), the most common primary malignant neoplasm of the liver. The causes of chronic liver inflammation include not only viral hepatitis but also chronic alcoholism, nonalcoholic steatohepatitis, and several other, less common, conditions^(1-3)^.

It is extraordinary to realize that with the technological advancement of the devices—mainly those of computed tomography (CT) and, especially, magnetic resonance imaging (MRI), through quantitative techniques and the various types of sequences available—radiologists have powerful noninvasive tools that function as "magnifying glasses" for the indirect study and precise evaluation of the cytoarchitecture of the organs of the body, with special attention to the liver. As detailed and demonstrated by Ramalho et al.^(4)^, in their article published in this issue of **Radiologia Brasileira**, MRI findings indirectly reproduce histopathological and hemodynamic changes, including altered hepatic morphology, fibrosis, and nodules, the spectrum of which ranges from regenerative nodules to dysplastic nodules of varying grades (from low to high) and, ultimately, HCC. The authors described the various advantages of MRI in relation to CT, including the fact that the former does not involve the use of ionizing radiation, has higher tissue resolution (which can facilitate the identification of intralesional fat components), and allows the use of extracellular and liver-specific contrast agents, as well as an excellent evaluation of the biliary tree by cholangiography. They also demonstrated the superiority of MRI over CT in the diagnosis of small HCCs, as well as in the detection of dysplastic nodules and diffuse HCC^(4)^.

Despite the importance of the abovementioned characteristics in the MRI sequences, the key tool for the diagnosis of HCC— and therefore for the evaluation of nodules in chronic liver diseases— is undoubtedly the study of the hemodynamic behavior against the background of the parenchyma, which can demonstrate the variation in the blood supply, identifying enhancement in the wash-in (arterial) phase and determining whether or not there is wash-out in the subsequent (portal and equilibrium) phases. In isolation, the most critical characteristic of HCC is the wash-in, which is attributed to the recruitment of new intratumoral arterioles and is highly sensitivity for the diagnosis of the disease^(5-8)^. However, high-grade dysplastic nodules and even arteriovenous shunts have this characteristic. Therefore, the combination of hypervascular enhancement in the arterial phase and wash-out in the later phases is highly specific for the diagnosis of HCC. However, if only the enhancement criterion were used, in 30-40% of HCC patients with cirrhosis would not be diagnosed with HCC, because they show atypical enhancement, especially in lesions smaller than 2.0 cm^(5)^. The differentiation, by imaging, among regenerative nodules, low-grade dysplastic nodules, high-grade dysplastic nodules, and HCC is fundamental for the proper management of the patient. As detailed in the study conducted by Ramalho et al.^(4)^, making those distinctions is feasible and reproducible through MRI examinations.

In a recent prospective study, Shankar et al.^(9)^ used a 3 T MRI scanner to obtain images of 20 patients, demonstrating not only that diffusion-weighted imaging (DWI) can be an alternative for detecting and characterizing HCC in patients with impaired renal function and allergic-type reactions to contrast medium but also that the use of the apparent diffusion coefficient (ADC) can be a useful noninvasive means of predict the degree of HCC differentiation. However, their results have yet to be confirmed by other groups.

The determination of signal intensity in T2-weighted sequences, the microscopic or macroscopic detection of fat by chemical shift techniques, the hemodynamic evaluation of tumors, the administration of extracellular or liver-specific contrast agents, and the use of quantitative techniques, such as DWI and determination of the ADC, are part of a true arsenal of tools and parameters that help us solve the puzzle that is the diagnosis of HCC^(2,4-10)^.

Radiologists are real modern-day detectives, with excellent "magnifying glasses". These lenses continue to be improved, allowing greater diagnostic precision, mainly in the evaluation of hemodynamics and of the cytoarchitecture of the tumor, thus eliminating the need for invasive methods in order to confirm the diagnosis. Our role as radiologists in the evaluation of hepatic nodules has undergone a major transformation due to advances in the quality of our "lenses", with a beneficial effect on the treatment of patients with chronic liver diseases. We must be attentive in order to keep pace with the great strides of these advances and embrace the future.
